# Intermittent Hypoxia Up-Regulates *CCL2*, *RETN,* and *TNFα* mRNAs in Adipocytes via Down-regulation of miR-452

**DOI:** 10.3390/ijms20081960

**Published:** 2019-04-22

**Authors:** Tomoko Uchiyama, Asako Itaya-Hironaka, Akiyo Yamauchi, Mai Makino, Sumiyo Sakuramoto-Tsuchida, Ryogo Shobatake, Hiroyo Ota, Maiko Takeda, Chiho Ohbayashi, Shin Takasawa

**Affiliations:** 1 Department of Biochemistry, Nara Medical University, 840 Shijo-cho, Kashihara, Nara 634-8521, Japan; uchiyama0403@naramed-u.ac.jp (T.U.); iasako@naramed-u.ac.jp (A.I.-H.); yamauchi@naramed-u.ac.jp (A.Y.); m.makino@naramed-u.ac.jp (M.M.); ssumiyo@naramed-u.ac.jp (S.S.-T.); rshobatake@naramed-u.ac.jp (R.S.); hiroyon@naramed-u.ac.jp (H.O.); 2 Department of Diagnostic Pathology, Nara Medical University, 840 Shijo-cho, Kashihara, Nara 634-8522, Japan; maikot@naramed-u.ac.jp (M.T.); ohbayashi@naramed-u.ac.jp (C.O.); 3 Second Department of Internal Medicine, Nara Medical University, 840 Shijo-cho, Kashihara, Nara 634-8522, Japan; 4 Department of Laboratory Medicine and Pathology, National Hospital Organization Kinki-chuo Chest Medical Center, 1180 Nagasone-cho, Kita-ku, Sakai, Osaka 591-8025, Japan

**Keywords:** adipokine, intermittent hypoxia, microRNA, sleep apnea syndrome

## Abstract

Sleep apnea syndrome (SAS), characterized by recurrent episodes of oxygen desaturation and reoxygenation (intermittent hypoxia [IH]), is a risk factor for insulin resistance. Recently, IH is considered to independently cause adipose tissue inflammation/dysfunction, leading to worsening insulin resistance; however, the detailed mechanism remains unknown. We exposed mouse 3T3-L1 and human SW872 adipocytes to experimental IH or normoxia for 24 h, and analyzed mRNA expression of several adipokines. We found that the mRNA levels of *RETN*, *TNFα*, and *CCL2* in SW872 and 3T3-L1 adipocytes were significantly increased by IH, whereas the promoter activities of these genes were not increased. A target mRNA search of microRNA (miR)s revealed that all human mRNAs have a potential target sequence for miR-452. The miR-452 level of IH-treated cells was significantly decreased compared to normoxia-treated cells. MiR-452 mimic and non-specific control RNA (miR-452 mimic NC) were introduced into SW872 cells, and the IH-induced up-regulation of the genes was abolished by introduction of the miR-452 mimic but not by the miR-452 mimic NC. These results indicate that IH stress down-regulates the miR-452 in adipocytes, resulting in increased levels of *RETN*, *TNFα*, and *CCL2* mRNAs, leading to insulin resistance in SAS patients.

## 1. Introduction

Sleep apnea syndrome (SAS) is a highly prevalent disease characterized by repetitive episodes of pharyngeal airway narrowing or obstruction during sleep, leading to apnea and hypopnea, often accompanied by a decrease in oxygen saturation [1]. It is a disorder affecting about 14–24% in men and 5–9% in women [2,3]. Accumulating evidence suggests that recurrent short cycles of oxygen desaturation followed by rapid reoxygenation (intermittent hypoxia [IH]), which are typical features of SAS, contribute to the development of impaired glucose tolerance/insulin resistance [4,5,6]. This relationship is considered to be irrelevant to the degree of obesity [7,8,9]. In addition to IH, several pathogenic mechanisms, such as sympathetic nervous system hyperactivity, oxidative stress, vascular endothelial dysfunction, and activation of the inflammatory cytokines, are all potential contributors to insulin resistance [10]. However, the detailed mechanisms by which IH induces insulin resistance in SAS patients are not well established. We have investigated how IH induces impaired insulin secretion/insulin resistance using pancreatic β cells, hepatocytes, and neuronal cells. We have reported that IH stress influences pancreatic β cell proliferation/dysfunction, hepatocytes proliferation/dysfunction, and synthesis of anorexigenic peptides in neuronal cells, which may lead to aggravate insulin resistance/type 2 diabetes [1,11,12].

Adipose tissue, complex tissue composed of preadipocytes, adipocytes, and stromal vascular cells, is one of the representative organs that contribute to worsening insulin resistance by inflammation and subsequent dysfunction [13]. Adipokines are hormones expressed and secreted from adipocytes in response to the systemic nutritional status, and some of which induce macrophage infiltration and inflammatory cytokine secretion [14,15]. Recently, it has been reported that SAS patient adipose tissues become inflamed by macrophage infiltration, inflammatory cytokines, and increased blood flow, resulting in insulin resistance [9,16]. Additionally, several animal studies suggested that IH induces adipocyte lipolysis, elevating plasma free fatty acid levels [17] and pro-inflammatory changes in adipose tissue independent of obesity [13], which may contribute to the pathogenesis of IH-induced insulin resistance. However, the experimental IH-induced change of adipokines secreted from preadipocytes/adipocytes is less clear. The phase of adipocyte differentiation (preadipocytes or adipocytes) in which such significant IH-induced changes of adipokines occur remains unknown. During differentiation, preadipocytes proliferate and become mature fat cells (adipocytes), which is a process of adipogenesis [18,19]. 

In the present study, using mouse and human adipocytes, we investigated changes of gene expression of several adipokines, synthesized/secreted from preadipocytes/adipocytes, in response to IH, as well as their regulatory mechanisms and found the expression of *resistin* (*Retn*), *tumor necrosis factor-α* (*Tnfα*), and *C-C motif chemokine ligand* 2 (*Ccl2*) was increased by IH via down-regulation of microRNA (miR)-452. 

## 2. Results

### 2.1. Gene Expression of RETN, TNFα, and CCL2 Was Increased by IH in Mouse 3T3-L1 Adipocytes and Human SW872 Cells 

**Figure 1:**
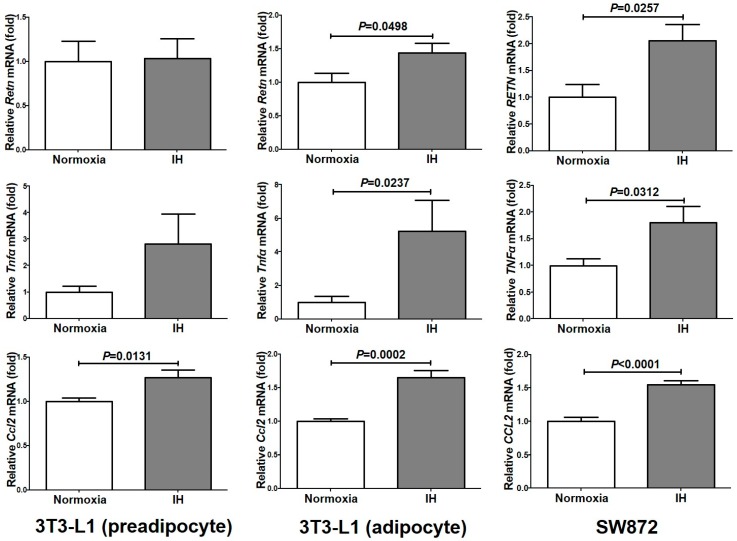
The mRNA levels of *Retn*, *Tnfα,* and *Ccl2* in 3T3-L1 cells (preadipocyte and adipocyte) and SW872 cells treated by normoxia or IH for 24 h. The levels of the adipokine mRNAs were measured by real-time RT-PCR using *Rig/RpS15* (in mouse) and *β-actin* (in human) as an endogenous control. Data is expressed as mean ± SE for each group (*n* = 4). The statistical analyses were performed using Student’s *t*-test.

We exposed mouse and human adipocytes (3T3-L1 preadipocytes, 3T3-L1 adipocytes, and SW872 cells) to normoxia or IH for 24 h. After the treatment, we measured the mRNA levels of several adipokines (*leptin* [*Lep*], *adiponectin* [*Adip*], *Retn*, *interleukin-6* (*IL-6*), *TNFα*, and *CCL2*) by real-time reverse transcription polymerase chain reaction (RT-PCR). As shown in Figure 1, the mRNA levels of *Ccl2* were significantly increased in 3T3-L1 preadipocytes (*P* = 0.0131), 3T3-L1 adipocytes (*P* = 0.0002), and SW872 cells (*P* < 0.0001). The mRNA levels of *Retn* and *Tnfα* were significantly increased in 3T3-L1 adipocytes (*P* = 0.0498 and *P* = 0.0237, respectively) and SW872 cells (*P* = 0.0257 and *P* = 0.0312, respectively).

**Figure 2:**
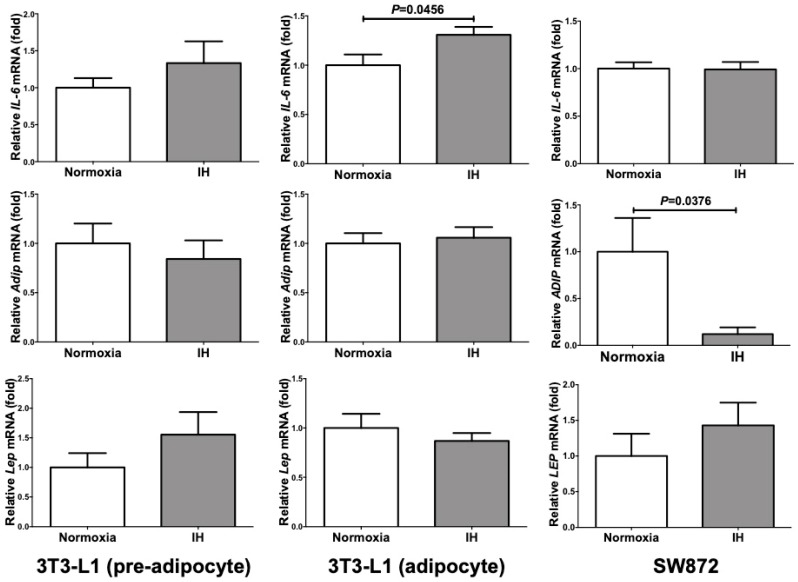
The mRNA levels of *Il-6*, *Adip*, and *Lep* in 3T3-L1 cells (pre-adipocyte and adipocyte) and SW872 cells treated by normoxia or IH for 24 h. The levels of the adipokine mRNAs were measured by real-time RT-PCR using *Rig/RpS15* (in mouse) and *β-actin* (in human) as an endogenous control. Data is expressed as mean ± SE for each group (*n* = 4). The statistical analyses were performed using Student’s *t*-test.

On the other hand, the mRNA levels of *IL-6* were significantly increased only in 3T3-L1 adipocytes (*P* = 0.0456), and unchanged in 3T3-L1 preadipocytes and SW872 cells (*P* = 0.3246 and *P* = 0.9366, respectively). *Adip* mRNA was significantly decreased in SW872 cells (*P* = 0.0376), and unchanged in either 3T3-L1 preadipocytes or adipocytes (*P* = 0.5787 and *P* = 0.7094, respectively). In all the analyzed cells, the mRNA levels of *Lep* were not significantly changed by IH (*P* = 0.2359 in 3T3-L1 preadipocytes, *P* = 0.4411 in 3T3-L1 adipocytes, and *P* = 0.3728 in SW872 cells) (Figure 2). Considering these results, IH stress might specifically up-regulate expression of *Retn*, *Tnfα* and *Ccl2* in 3T3-L1 adipocytes and SW872 cells.

**Figure 3:**
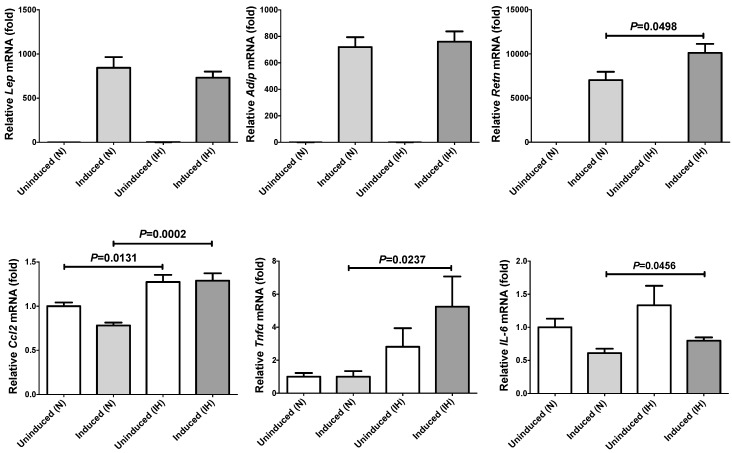
The mRNA levels of *Lep*, *Adip*, *Retn*, *Ccl2*, *Tnfα*, and *IL-6* in 3T3-L1 cells treated by normoxia or IH for 24 h. Figure shows relative comparison of mRNA expression in 3T3-L1 preadipocytes with 3T3-L1 adipocytes. The levels of the adipokine mRNAs were measured by real-time RT-PCR using *Rig/RpS15* as an endogenous control. Data are expressed as mean ± SE for each group (*n* = 4). The statistical analyses were performed using Student’s *t*-test.

The mRNA levels of *Lep*, *Adip* and *Retn* in 3T3-L1 adipocytes (normoxia) were significantly increased compared to 3T3-L1 preadipocytes (884.0 ± 120.9 fold [*P* < 0.0001], 719.2 ± 74.96 fold [*P* < 0.0001], and 7,032 ± 947.5 fold [*P* < 0.0001], respectively), suggesting that these genes are expressed specifically after differentiation to adipocytes. In contrast, *Ccl2*, *Tnfα* and *IL-6*, expressed in many different types of cell, were not significantly increased but rather decreased after differentiation from preadipocytes to adipocytes (0.781 ± 0.0329 fold [*P* = 0.0021], 0.639 ± 0.296 fold [*P* = 0.4242], and 0.610 ± 0.0664 fold [*P* = 0.0233], respectively; Figure 3). 

We are unable to explain the exact reason why there is a decrease in *Ccl2*, *Tnfα*, and *IL-6* expression compared to *Lep*, *Adip*, and *Retn*. However, there are some clues that may help explain. (1) Tnfα treatment decreased *Adip* mRNA expression in 3T3-L1 cells [20]. (2) Tnfα also works as a potent negative regulator for *Retn* mRNA expression in 3T3-L1 cells [21,22]. (3) In addition, Tnfα potently induced *IL-6* mRNA expression in 3T3-L1 cells [23]. (4) IL-6 works as a negative regulator for *Adip* mRNA expression in 3T3-L1 cells [24]. In our experiments, *Tnfα* mRNA expression was unchanged during 3T3-L1 cell differentiation. Thus, the down-regulation of *Adip* and *Retn* mRNAs by Tnfα and up-regulation of IL-6 mRNA by Tnfα did not work. As a result, up-regulation of *Adip* and *Retn* mRNAs were clearly detected. In addition, as the IL-6 expression was unchanged, down-regulation of *Adip* mRNA did not occur.

**Figure 4:**
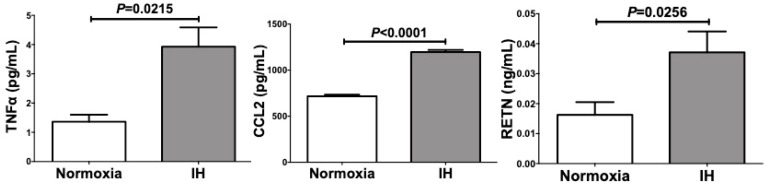
Concentrations of RETN, TNFα, and CCL2 in SW872 cell culture medium were measured by ELISA. SW872 cells were treated by normoxia or IH for 24 h. Data are expressed as means ± SE for each group (*n* = 3).

We further measured RETN, TNFα, and CCL2 protein in the culture medium by ELISA and found that the levels of RETN (*P* = 0.0256), TNFα (*P* = 0.0215), and CCL2 (*P* < 0.0001) were significantly increased by IH in SW872 cells (Figure 4).

### 2.2. The Promoter Activities of RETN, TNFα, and CCL2 Were not Increased by IH

**Figure 5:**
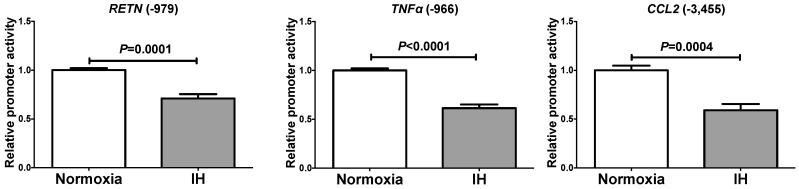
Luciferase assays of promoter activities of *RETN*, *TNFα*, and *CCL2* in SW872 cells. Reporter plasmids prepared by inserting the promoter fragments of *RETN* (−979~+20), *TNFα* (−966~+19), and *CCL2* (−3455~+25), upstream of a firefly luciferase reporter gene in pGL4.17 vector were transfected into SW872 cells. After cells were exposed either to IH or normoxia for 24 h, the cells were lysed and the promoter activities of *RETN*, *TNFα*, and *CCL2* were measured. All data are represented as the mean ± SE of the samples (*n* = 5–6). The statistical analyses were performed using Student’s *t*-test.

To determine whether the IH-induced increases in *RETN*, *TNFα* and *CCL2* mRNAs were caused by activation of transcription, a 999 bp fragment containing 979 bp of the *RETN* promoter, a 985 bp fragment containing 966 bp of the *TNFα* promoter, and a 3480 bp fragment containing 3455 bp of the *CCL2* promoter were fused to the luciferase gene of pGL4.17 and transfected into SW872 cells. After IH stimulation, we measured promoter activities and found that *RETN*, *TNFα*, and *CCL2* promoter activities were significantly decreased by IH in SW872 cells (Figure 5). These results suggested that the gene expression of *RETN*, *TNFα*, and *CCL2* in response to IH was not regulated by transcription. 

### 2.3. The MiR-452 Level Was Significantly Decreased by IH

**Figure 6:**
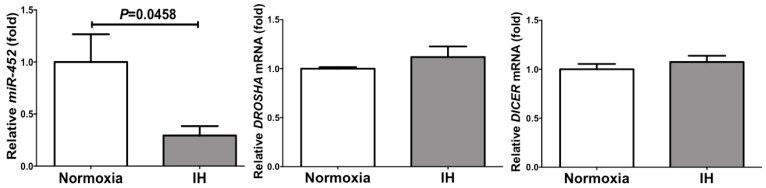
The levels of *miR-452*, *DROSHA* mRNA, and *DICER* mRNA of SW872 cells treated with normoxia or IH for 24 h. The levels of miR-452 and *DROSHA* and *DICER* mRNAs were measured by real-time RT-PCR using *U6* (for miR-452) and *β-actin* (for *DROSH* and *DICER*) as an endogenous control. Data are expressed as mean ± SE for each group (*n* = 4–6). The statistical analyses were performed using Student’s *t*-test.

We considered a possible explanation that the IH-induced up-regulation of *RETN*, *TNFα*, and *CCL2* was controlled post-transcriptionally. Therefore, we searched targeted miRNA using the MicroRNA.org program (http://www.microrna.org/microrna/home.do), which revealed that *RETN*, *TNFα*, and *CCL2* mRNAs have a potential target sequence for miR-452. There were no other miRNA candidates targeting all three genes. We measured the miR-452 levels of IH-treated cells by RT-PCR and found that the level was significantly lower than that of normoxia-treated cells (0.02937 ± 0.09028 fold vs normoxia, *P* = 0.0458). The possible reasons as to why the level of miR-452 was decreased by IH include; mRNA levels of some enzymes involved in miRNA biosynthesis/degradation are influenced by IH; and the level of miR-452 was specifically decreased by IH either via decreased biosynthesis or enhanced degradation. We measured the mRNA levels of *ribonuclease type III* (*DROSHA*) and *endoribonuclease Dicer* (*DICER*), which are involved in the biosynthesis of miRNAs [25,26] and found that their expression was unchanged by IH (Figure 6). These results suggest that miR-452 plays a key role in post-transcriptional regulation of mRNA levels of *RETN*, *TNFα*, and *CCL2*. To investigate whether *RETN*, *TNFα,* and *CCL2* expression in IH is regulated by miR-452, miR-452 mimic and non-specific control RNA (miR-452 mimic NC) were introduced into SW872 cells with IH/normoxia exposure, and the mRNA levels of *RETN*, *TNFα*, and *CCL2* were measured by real-time RT-PCR. 

**Figure 7:**
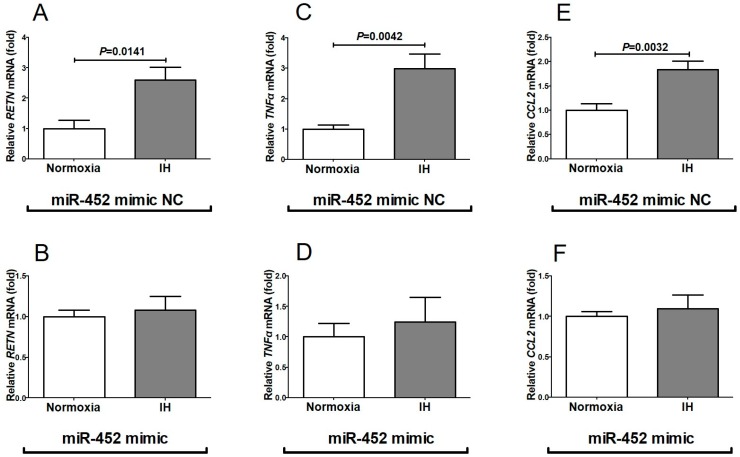
Effects of miR-452 mimic transfection on *RETN*, *TNFα*, and *CCL2* expression. The miR-452 mimic (5′-AACUGUUUGCAGAGGAAACUG-3′, 5′-GUUUCCUCUCUGCAAACAGUUUU-3′) and non-specific control RNA (miR-452 mimic NC) (5′-UUCUCCGAACGUGUCACGUtt-3′, 5′-ACGUGACACGUUCGGAGAAtt-3′) were synthesized by Nihon Gene Research Laboratories, Inc. (NGRL; Sendai, Japan) and introduced into SW872 cells using Lipofectamine^®^ RNAiMAX just before IH/normoxia exposure, and the mRNA levels of *RETN*, *TNFα*, and *CCL2* were measured by real-time RT-PCR, as described in Materials and Methods. The expression of *RETN*, *TNFα*, and *CCL2* mRNA was measured by real-time RT-PCR using *β-actin* as an endogenous control. Figure represents (**A**) *RETN* mRNA expression in miR-452 mimic NC-introduced cells, (**B**) *RETN* mRNA expression in miR-452 mimic-introduced cells, (**C**) *TNFα* mRNA expression in miR-452 mimic NC-introduced cells, (**D**) *TNFα* mRNA expression in miR-452 mimic-introduced cells, (**E**) *CCL2* mRNA expression in miR-452 mimic NC-introduced cells, and (**F**) *CCL2* mRNA expression in miR-452 mimic-introduced cells. Data are expressed as mean ± SE for each group (*n* = 6). The statistical analyses were performed using Student’s *t*-test.

As shown in Figure 7, we found that the IH-induced increases in *RETN*, *TNFα* and *CCL2* mRNAs were abolished by the introduction of miR-452 mimic but not by miR-452 mimic NC. These findings indicate that IH stress down-regulates the miR-452 level in human adipocytes (Figure 6) and that the levels of *RETN*, *TNFα*, and *CCL2* mRNAs are increased via the miR-452 mediated mechanism.

## 3. Discussion

In this study, we demonstrated that IH exposure induced increases of *RETN*, *TNFα*, and *CCL2* mRNA levels. We further studied that the mechanisms by which IH up-regulates the mRNA levels of adipokines such as *RETN*, *TNFα*, and *CCL2*, and found the possibility of post-transcriptional miRNA-regulated mechanisms. 

Causal mechanisms mediating the association between IH and insulin resistance/glucose intolerance are not well established; however, augmented dysfunction/inflammation in adipose tissue might be involved [9,13,16,17,27,28]. It is well known that macrophages, which infiltrate into adipose tissue, increase in obese patients, resulting in up-regulation of pro-inflammatory cytokines, such as TNFα and IL-6 [29,30]. However, Thorn et al. reported that adipose tissue is influenced by hypoxia in SAS patients independent of obesity [16]. Some mechanisms linking IH stress and adipose tissue inflammation have been established in 3T3-L1 cells and mouse models [13,17,27]. Recently, IH was shown to induce impairment of adipose tissue, leading to various changes in secretion of inflammatory cytokines, called adipokines [16,17,28]. Adipokines, which are bioactive mediators produced and released from adipocytes, play important roles in many physiological and pathophysiological processes that contribute to modulate homeostasis, lipid and/or glucose metabolism, blood pressure, inflammation, and atherosclerosis [14,29].

Ccl2, also referred to as monocyte chemoattractant protein-1, is a key regulator of monocyte infiltration of adipose tissue that plays a central role in the development and maintenance of chronic adipose tissue inflammation and insulin resistance [14,31,32]. In this study, the mRNA levels of *Ccl2* were significantly increased in the IH condition in mouse 3T3-L1 cells (preadipocytes and adipocytes), and human SW872 cells. TNFα is a pro-inflammatory cytokine mainly produced by monocytes and macrophages. TNFα plays a key role in obesity-related insulin resistance, and increased TNFα levels contribute to impaired glucose homeostasis; however, the role of TNFα as an adipokine in the IH condition has not been fully elucidated [14,31]. In this study, TNFα was produced/secreted from 3T3-L1 adipocytes and SW872 cells in the IH condition. In SAS patients, the elevation of circulating levels of TNFα and CCL2 was reported [33,34]. Some researchers reported that TNFα and CCL2 production in monocytes was up-regulated in SAS patients [35,36], and Tnfα induced the mRNA expression of *Ccl2* and *IL-6*, and attenuated *Adip* mRNA in 3T3-L1 cells [37]. However, as the expression of *Adip* mRNA in 3T3-L1 cells was unchanged despite significant increases of Tnfα and the up-regulation of IL-6 in differentiated 3T3-L1 being small, the up-regulation of adipokine mRNAs (*Tnfα*, *Ccl2*, and *Retn*) could be independently and directly caused by IH. There are few reports about expression of *TNFα* and *CCL2* genes in adipocytes of SAS patients or under the experimental IH condition. From our results, the expression of *TNFα* and *CCL2* was increased in adipocytes and TNFα and CCL2 could act as adipokines contributing to worsening insulin resistance in the IH condition. 

Resistin is a pro-inflammatory adipokine and initially named because of its relationship to insulin resistance in rodents [31,38]. Although resistin expression is restricted to adipocytes in mice, it was reported to be produced mainly by macrophages and monocytes in humans. However, Yin et al. reported that resistin was overexpressed in the late stage of adipogenesis of SW872 cells [39]. Recently, resistin has been proposed as a marker of cardiovascular diseases [40,41] and suggested to have a possible link to SAS [42]; however, the relation between insulin resistance and resistin in humans remains unclear. Our results suggested that Retn was produced/secreted from 3T3-L1 adipocytes and SW872 cells in the IH condition. In addition, recent studies indicated that transcription of the *Retn* gene is induced by pro-inflammatory cytokines such as IL-1, IL-6, and TNFα, and that Retn promotes the expression of *TNFα* and *IL-6* by macrophages and monocytes [31]. Considering our results and these findings, *RETN* may be up-regulated in SAS patients and can lead them to insulin resistance/type 2 diabetes. Adding to *Ccl2* and *Tnfα*, *Retn* may be one of the adipokines increasing insulin resistance in IH.

Adip is specifically expressed and secreted from adipocytes. Unlike most other adipokines, the level of plasma ADIP in humans is negatively correlated with adiposity, insulin resistance, and type 2 diabetes. A causal role of ADIP in the development of type 2 diabetes, dyslipidemia, and cardiovascular diseases has been established; however, the influence of the IH condition remains unknown [29,43,44]. In our study, the mRNA level of *ADIP* was significantly decreased by IH in SW872 cells, while it was not changed in differentiated or undifferentiated 3T3-L1 cells. One of the reasons that *ADIP* mRNA levels decrease in SW872 cells is the interaction of *ADIP* and *TNFα*; that is, in the IH condition, *TNFα* may suppress ADIP synthesis or activity [14]. IH-induced ADIP decrease may relate to worsening insulin resistance/type 2 diabetes in SAS patients; therefore, further in vivo studies will be needed. 

We investigated the mechanisms by which IH up-regulates the mRNA levels of *RETN*, *TNFα*, and *CCL2*, and found that the promoter activities of the genes were not increased by IH. This suggests that IH-induced up-regulation of *RETN*, *TNFα*, and *CCL2* mRNAs is regulated in the post-transcriptional step. MiRNAs are small non-coding RNAs, ~22 nucleotides in length, which modulate gene expression either by translational suppression or degradation of mRNA through binding to the 3′-untranslated regions of target genes in a base-paring manner [45]. They affect the stability of their target mRNAs, resulting in changes in the amount of target mRNA, which is one of the mechanisms of post-transcriptional regulation. Until now, some studies about the role of miR-452 have been performed in malignant neoplasms such as pancreatic cancer [46], glioma [47], lung cancer [48,49], and breast cancer [50]. A number of studies have indicated that miRNAs play roles in the regulation of many biological processes (migration, metastasis, cell proliferation, apoptosis, chemosensitivity, etc.) for these various types of tumors. MiRNA studies in type 2 diabetes reported a correlation between circulating miRNAs and type 2 diabetes [51,52]. However, the papers did not indicate an involvement of miR-452 in type 2 diabetes/insulin resistance. In the area of gestational diabetes, miR-452 was reported to be upregulated in human umbilical endothelial cells (HUVEC) from infants of mothers with gestational diabetes [53]. TNFα induced oxidative stress [54] and inflammatory gene expression [55] in HUVEC, and oxidative stress induced up-regulation of CCL2 [56]. In addition, RETN was reported to induce HUVEC dysfunction [57]. The up-regulation of miR-452 in HUVEC from infants of mothers with gestational diabetes might be a defense mechanism to down-regulate TNFα, CCL2, and RETN in HUVEC.

Little is known about the mechanisms regulating adipose tissue inflammation; however, accumulating evidence indicates the importance of the roles of miRNAs in cholesterol and lipid metabolism, and controlling insulin signaling [58]. In the last decade, miRNAs have emerged as key epigenetic regulators in the adipocyte development process and functions [15]. In this study, miR-452 with common target sequence in *RETN*, *TNFα*, and *CCL2* mRNAs could contribute to worsening glucose intolerance [59,60,61] in the IH-condition by up-regulation of *RETN*, *TNFα*, and *CCL2* mRNAs. 

In conclusion, this study revealed that the gene expression of *RETN, TNFα*, and *CCL2* were increased via down-regulation of the miR-452 level in IH-treated adipocytes. It is suggested that, in SAS patients, up-regulation of *RETN*, *TNFα*, and *CCL2* may induce a pro-inflammatory phenotype of the adipose tissue, leading to the development of insulin resistance and decreased insulin sensitivity, and miR-452 could play crucial roles in regulation of these gene expressions. 

## 4. Materials and Methods 

### 4.1. Cell Culture

Mouse 3T3-L1 cells were purchased from the National Institutes of Biomedical Innovation, Health and Nutrition, JCRB Cell Bank (Ibaraki, Japan) and human liposarcoma SW872 cells were purchased from American Type Culture Collection (Manassas, VA, USA). 3T3-L1 and SW872 cells were grown in DMEM (Wako Pure Chemical Industries, Ltd., Osaka, Japan) containing 10% (*v*/*v*) fetal calf serum (FCS), 100 units/mL penicillin G (Wako) and 100 μg/mL streptomycin (Wako) as described [62]. Cells were exposed to either normoxia (21% O_2_, 5% CO_2_, and balanced N_2_) or intermittent hypoxia (IH: 64 cycles of 5 min sustained hypoxia [1% O_2_, 5% CO_2_, and balanced N_2_] and 10 min normoxia) using a custom-designed, computer-controlled incubation chamber attached to an external O_2_–CO_2_–N_2_ computer-driven controller (O_2_ programmable control, 9200EX, Wakenyaku Co., Ltd., Kyoto, Japan), as described [1,11,12,63]. We used this in vitro model of IH, resulting in fluctuations of pressure of oxygen similar to the IH condition in patients with a severe degree of SAS, repeatedly exposed to severe hypoxemia followed by mild hypoxemia or normoxia (i.e., IH) [9]. Kimura and co-workers previously reported that the magnitude of IH expressed by peripheral oxygen saturation (SpO_2_) fluctuated between 75–98% and 50–80% in SAS [1,64], which was almost equivalent to the medium condition in the present study.

### 4.2. Differentiation of 3T3-L1 Cells into Adipocyte-Like Cells

Insulin, methylisobutylxanthine (IBMX) and dexamethasone were purchased from Sigma-Aldrich (St. Louis, MO, USA). To induce adipocyte differentiation, (1) cells were cultured in DMEM medium for 2 days, (2) treated with DMEM medium containing 0.5 mM IBMX, 1 μM dexamethasone and 10 μg/mL insulin for 3 days, and finally (3) cultured with DMEM medium containing 10 µg/mL insulin for 3 days, as described [65]. The cells were then incubated in fresh DMEM medium for an additional 2 days and fully differentiated adipocyte-like cells were obtained. 

### 4.3. RT-PCR

Total RNA was isolated using a RNA protect cell mini kit (Qiagen, Hilden, Germany) from 3T3-L1 cells (preadipocytes and adipocytes) and SW872 cells, and cDNA was synthesized from total RNA as template using a High Capacity cDNA Reverse Transcription kit (Applied Biosystems, Foster City, CA) as described [11,12,63,66,67,68,69,70,71]. Real-time polymerase chain reaction (PCR) was performed using SYBR^®^ Fast qPCR kit (KAPA Biosystems, Boston, MA) and a Thermal Cycler Dice Real Time System (Takara, Kusatsu, Japan). All the PCR primers were synthesized by Nihon Gene Research Laboratories, Inc. (NGRL; Sendai, Japan), and the primer sequences for each primer set are described in Table 1. PCR was performed with an initial step of 3 min at 95 °C followed by 40 cycles of 3 s at 95 °C and 20 s at 60 °C for *β-actin*; 45 cycles of 10 s at 95 °C, 5 s at 60 °C and 20 s at 72 °C for *rat insulinoma gene* (*Rig*)*/ribosomal protein S15* (*RpS15*); 45 cycles of 3 s at 95 °C and 20 s at 62 °C for *TNFα* (human) and *IL-6* (human); 45 cycles of 3 s at 95 °C and 20 s at 60 °C for *resistin* (*RETN*, human and mouse), *C-C motif chemokine ligand 2* (*CCL2*, human and mouse), *adiponectin* (*ADIP*, human and mouse), *IL-6* (mouse), *leptin* (*LEP*, human and mouse), *TNFα* (mouse), *ribonuclease type III* (*DROSHA*), *endoribonuclease Dicer* (*DICER*), and *microRNA-452* (*miR-452*). The mRNA expression levels were normalized to the mRNA level of *Rig/RpS15* in mouse samples or *β-actin* in human samples, and the *miR-452* level was normalized to the *U6* RNA level.

### 4.4. Measurement of RETN, TNFα and CCL2 in Culture Medium by Enzyme-Linked Immunosorbent Assay (ELISA)

Cells were exposed to either normoxia or IH for 24 h, culture medium was collected, and the concentration of RETN, TNFα, and CCL2 was measured by using a Human Resistin (RETN) ELISA kit (R&D Systems, Minneapolis, MN), Human TNFα ELISA kit (Diaclone SAS, Besançon, France) and Human C-C motif chemokine ligand 2 (CCL2) ELISA kit (R&D Systems) according to the instructions of the suppliers.

### 4.5. Construction of Reporter Plasmid and Luciferase Assay

Reporter plasmids were prepared by inserting the promoter fragments of human *RETN* (−979~+20), *TNFα* (−966~+19), and *CCL2* (−3455~+25) upstream of a firefly luciferase reporter gene in the pGL4.17 vector (Promega, Madison, WI). The reporter plasmids were transfected into human SW872 adipocytes using Lipofectamine^®^ 3000 (Invitrogen, Waltham, MA), as described [68,69,70], and the cells were exposed to either 64 cycles/24 h of IH, mimicking adipocytes of SAS patients, or normoxia for 24 h. After cells were exposed to IH, the cells were harvested and cell extracts were prepared in extraction buffer (0.1 M potassium phosphate, pH 7.8/0.2% Triton X-100; Life Technologies, Carlsbad, CA, USA). To monitor transfection efficiency, pCMV-SPORT-βgal plasmid (Life Technologies) was co-transfected in all experiments at a 1:10 dilution. Luciferase activity was measured using a PicaGene luciferase assay system (Toyo-ink, Tokyo, Japan) and was normalized by the β-galactosidase activity as described previously [11,12,62,63,67,68,69,70,71,72,73,74].

### 4.6. MiRNA Extraction, Reverse Transcription, and Real-Time Quantitative PCR

Total RNA including miRNA was isolated from SW872 cells using the miRNeasy mini kit (Qiagen) according to the manufacturer’s instructions. An equal amount of DNase-treated RNA was Poly-A tailed using a Mir-X^TM^ miRNA first strand synthesis kit (Clontech Laboratories, Inc., Mountain View, CA, USA) according to the manufacturer’s protocol. The condition for PCR was 95 °C for 10 s, followed by 45 cycles of amplification (95 °C, 5 s, 60 °C, 20 s). U6 small nuclear RNA was used as an endogenous control for miRNA as described [11,71,73]. The primers are listed in Table 1.

### 4.7. MiR-452 Mimic Transfection

MiR-452 mimic (5′-AACUGUUUGCAGAGGAAACUG-3′, 5′-GUUUCCUCUCUGCAAACAGUUUU-3′) and non-specific control RNA (miR-452 mimic NC) (5′-UUCUCCGAACGUGUCACGUtt-3′, 5′-ACGUGACACGUUCGGAGAAtt-3′) were synthesized by NGRL and introduced into SW872 cells using Lipofectamine^®^ RNAiMAX (Invitrogen) [11,67,68,69,70,71,73] just before IH/normoxia exposure, and the mRNA levels of *RETN*, *TNF-α*, and *CCL2* were measured by real-time RT-PCR, as described [11,12,63,66,67,68,69,70,71,73,74].

### 4.8. Data Analysis

Results are expressed as mean ±SE. Statistical significance was determined by Student’s *t*-test using GraphPad Prism software (GraphPad Software, La Jolla, CA, USA).

## Figures and Tables

**Table 1:** Primers used for real-time RT-PCR.

Target mRNA/miR	Primer Sequence
Mouse *Adip* (NM_009065)	5′-GGCTCTGTGCTGCTCCATCT-3′
	5′-AGAGTCGTTGACGTTATCTGCATAG-3′
Mouse *Retn* (NM_022984)	5′-GTACCCACGGGATGAAGAACC-3′
	5′-GCAGAGCCACAGGAGCAG-3′
Mouse *IL-6* (NM_031168)	5′-ACAACCACGGCCTTCCCTACTT-3′
	5′-CAGGATTTCCCAGCGAACATGTG-3′
Mouse *TNFα* (NM_013693)	5′-CCTCCCTCTCATCAGTTCTA-3′
	5′-ACTTGGTGGTTTGCTACGAC-3′
Mouse *Ccl2* (NM_011333)	5′-CCACTCACCTGCTGCTACTCAT -3′
	5′-TGGTGATCCTCTTGTAGCTCTCC -3′
Mouse *Rig/RpS15* (NM_009091)	5′-ACGGCAAGACCTTCAACCAG-3
	5′-ATGGAGAACTCGCCCAGGTAG-3′
Human *ADIP* (NM_001177800)	5′-CATGACCAGGAAACCACGACT -3′
	5′-TGAATGCTGAGCGGTAT -3′
Human *RETN* (NM_020415)	5′-TCCTCCTCCTCCCTGTCCTGG-3′
	5′-CAGTGACATGTGGTCTGGGCG -3′
Human *IL-6* (NM_000600)	5′-GGTACATCCTCGACGGCATC-3′
	5′-GCCTCTTTGCTGCTTTCACAC-3′
Human *TNFα* (NM_000594)	5′-CTTCTCCTTCCTGATCGTGG-3′
	5′-TCTCAGCTCCACGCCATT-3′
Human *CCL2* (NM_002982)	5′-GTCTCTGCCGCCCTTCTGT-3′
	5′-TTGCATCTGGCTGAGCGAG -3′
Human *DICER* (NM_177438)	5′-GAGCTGTCCTATCAGATCAGGG-3′
	5′-ACTTGTTGAGCAACCTGGTTT-3′
Human *DROSHA* (NM_013235)	5′-GGCCCGAGAGCCTTTTATAG-3′
	5′-TGCACACGTCTAACTCTTCCAC-3′
Human *β-actin* (NM_001101)	5′-GCGAGAAGATGACCCAGA-3′
	5′-CAGAGGCGTACAGGGATA-3′
Human *miR-452* (NR_029973)	5′-GCGAACTGTTTGCAGAGG-3′
	5′-CAGTGCGTGTCGTGGAGT-3′
Human *U6* (NR_004394)	5′-CTCGCTTCGGCAGCACA-3′
	5′-AACGCTTCACGAATTTGCGT-3′

## Abbreviations

ADIP	Adiponectin
CCL2	C-C motif chemokine ligand 2
DICER	Endoribonuclease Dicer
DROSHA	Ribonuclease type III
ELISA	Enzyme-linked Immunosorbent assay
FCS	Fetal calf serum
HUVEC	Human umbilical endothelial cells
IBMX	methylisobutylxanthine
IH	Intermittent hypoxia
IL-6	Interleukin-6
LEP	Leptin
miRNA	MicroRNA
RETN	Resistin
RpS15	Ribosomal protein S15
RT-PCR	Reverse transcriptase-polymerase chain reaction
SAS	Sleep apnea syndrome
TNFα	Tumor necrosis factor-α
